# The acyl-activating enzyme PhAAE13 is an alternative enzymatic source of precursors for anthocyanin biosynthesis in petunia flowers

**DOI:** 10.1093/jxb/erw426

**Published:** 2016-12-07

**Authors:** Guoju Chen, Heping Liu, Qian Wei, Huina Zhao, Juanxu Liu, Yixun Yu

**Affiliations:** 1 Guangdong Key Laboratory for Innovative Development and Utilization of Forest Plant Germplasm, College of Forestry and Landscape Architecture, South China Agricultural University, Guangzhou 510642, China; 2 College of Horticulture, South China Agricultural University, Guangzhou 510642, China

**Keywords:** AAE13, anthocyanin synthesis, malonic acid, malonyl-CoA, petunia.

## Abstract

Anthocyanins, a class of flavonoids, are responsible for the orange to blue coloration of flowers and act as visual attractors to aid pollination and seed dispersal. Malonyl-CoA is the precursor for the formation of flavonoids and anthocyanins. Previous studies have suggested that malonyl-CoA is formed almost exclusively by acetyl-CoA carboxylase, which catalyzes the ATP-dependent formation of malonyl-CoA from acetyl-CoA and bicarbonate. In the present study, the full-length cDNA of *Petunia hybrida acyl-activating enzyme 13* (*PhAAE13*), a member of clade VII of the *AAE* superfamily that encodes malonyl-CoA synthetase, was isolated. The expression of *PhAAE13* was highest in corollas and was down-regulated by ethylene. Virus-induced gene silencing of petunia *PhAAE13* significantly reduced anthocyanin accumulation, fatty acid content, and cuticular wax components content, and increased malonic acid content in flowers. The silencing of *PhAAE3* and *PhAAE14*, the other two genes in clade VII of the *AAE* superfamily, did not change the anthocyanin content in petunia flowers. This study provides strong evidence indicating that PhAAE13, among clade VII of the *AAE* superfamily, is specifically involved in anthocyanin biosynthesis in petunia flowers.

## Highlight

The silencing of petunia *PhAAE13,* encoding malonyl-CoA synthetase, significantly reduced anthocyanin accumulation and increased the malonic acid content in flowers, suggesting the involvement of PhAAE13 in anthocyanin biosynthesis in petunia.

## Introduction

**Fig. 1.**
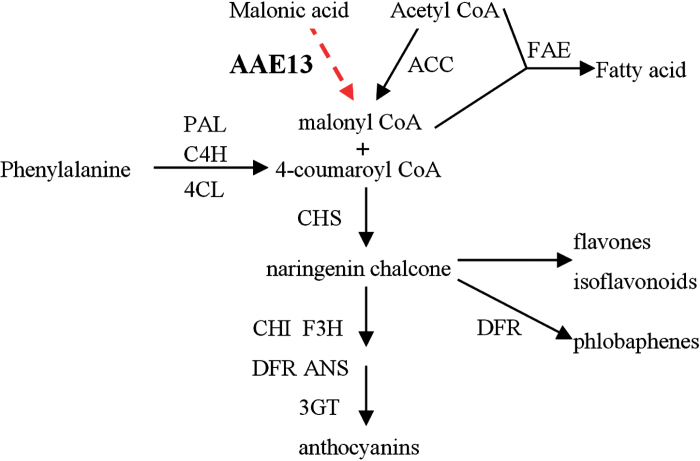
A simplified view of the anthocyanin biosynthesis pathway (Koes *et al.*, 2005; Rausher *et al.*, 1999). 3GT, UDP-glucose:flavonoid 3-*O*-glycosyl transferase; 4CL, 4-coumarate:CoA ligase; AAE13, acyl-activating enzyme 13 (malonyl-CoA synthetase); ACC, acetyl-CoA carboxylase; ANS, anthocyanin synthase; C4H, cinnamate-4-hydroxylase; CHS, chalcone synthase; CHI, chalcone flavanone isomerase; DFR, dihydroflavonol 4-reductase; F3H, flavanone 3β-hydroxylase; FAE, fatty acid elongase; PAL, phenylalanine ammonia-lyase.

Flavonoids are secondary metabolites that are common to all higher plants. This category of compounds includes anthocyanins, flavanones, flavones, and flavonols (Koes *et al.*, 1994). Anthocyanin pigments provide flowers with bright red and blue colors, and are induced in vegetative tissues by various signals (Mol *et al.*, 1996). The anthocyanin biosynthetic pathway likely represents one of the best-studied examples of higher plant secondary metabolism (Fig. 1) (Koes *et al.*, 2005; Rausher *et al.*, 1999).

The genes encoding flavonoid enzymes have been isolated from a variety of plant species. In plants, flavonoids are formed by adding three molecules of malonyl-CoA to a coumaroyl-CoA starter, which is catalyzed by chalcone synthase and forms naringenin chalcone (Halls and Yu, 2008). Malonyl-CoA is the precursor for the formation of flavonoids and anthocyanins (Peer *et al.*, 2001). It has been generally accepted that malonyl-CoA is formed almost exclusively by acetyl-CoA carboxylase (ACC; EC 6.4.1.2), which catalyzes the ATP-dependent formation of malonyl-CoA from acetyl-CoA and bicarbonate (Belkebir and Benhassaine-Kesri, 2013; Fatland *et al.*, 2005).

In addition to ACCs, malonyl-CoA synthetase ligates malonic acid and CoA to generate malonyl-CoA directly in plants. Arabidopsis acyl-activating enzyme 13 (AtAAE13), a member of the clade VII AAE superfamily, was identified as a malonyl-CoA synthetase (Chen *et al.*, 2011; Shockey and Fulda, 2003). Because *aae13*-null mutants grow poorly and accumulate malonic acid, AAE13 has been implicated in the detoxification of short-chain organic acids (Chen *et al.*, 2011). The overexpression of *AtAAE13* in *Saccharomyces cerevisiae* simultaneously increased lipid and resveratrol accumulation, and AAE13 partially complemented the temperature-sensitive acc1 mutant, replacing this key enzyme in central metabolism (Wang *et al.*, 2014). Recently, Guan and Nikolau (2016) suggested that *Arabidopsis* AAE13 has two isoforms, translated from two types of transcripts—one that contains a mitochondrial-targeting pre-sequence, and one that does not—that are localized in both cytosol and mitochondria. In addition, bacterial malonyl-CoA synthetase has been used in fatty acid and flavonoid biosynthesis (Leonard *et al.*, 2008; Park *et al.*, 2011). However, it remains unknown whether the AAE13 pathway, which catalyzes the synthesis of malonyl-CoA from malonic acid, is involved in anthocyanin biosynthesis.

The petunia flower has served as a model for the study of flavonoid or anthocyanin synthesis (Gerats and Vandenbussche, 2005). More than 10 genes encoding enzymes and transcription regulators involved in anthocyanin synthesis, such as ANTHOCYANIN2 (AN2), AN1, and AN11, have been identified (Koes *et al.*, 2005). The blue petal color of petunia *ph1* mutants reflects a failure to hyperacidify vacuoles, and *PH1* encodes a P3BATPase, hitherto known as a Mg^2+^ transporter in bacteria only, which resides in the vacuolar membrane (tonoplast) (Faraco *et al.*, 2014).

In the present study, petunia *PhAAE13* full-length cDNA was isolated. The expression of *PhAAE13* was decreased by ethylene treatment and increased by ultraviolet B (UV-B) radiation. Virus-induced gene silencing (VIGS) of *PhAAE13* significantly reduced anthocyanin accumulation and increased malonic acid accumulation in petunia. These results indicate that the new structural gene *PhAAE13* plays an important role in anthocyanin biosynthesis.

## Materials and methods

### Plant materials

*Petunia hybrida* ‘Ultra’ plants were grown under greenhouse conditions (22–25 °C, 14 h light/10 h dark) as described by Yang *et al.* (2015). Eight to ten petunia flowers were harvested at anthesis (corollas 90° reflexed) and immediately placed in tap water. The stems, leaves, and roots were collected from plants at the vegetative stage when the plants were ~25 cm in height. All tissues were frozen in liquid nitrogen and stored at –80 °C until used for RNA extraction. The fresh weights were measured immediately before freezing (Yang *et al.*, 2015). All experiments were conducted at least three times with independently collected and extracted tissues unless otherwise noted.

### RNA extraction, RT-PCR, and cloning of the petunia *PhAAE13, PhAAE3*, *PhAAE14*, *PhACC1*, and *PhACC2* genes

Total RNA was isolated and reverse transcribed according to the methods of Liu *et al.* (2011). *PhAAE13, PhAAE3*, *PhAAE14*, and *PhACC* cDNAs were cloned according to previously described protocols (Yu *et al.*, 2011). Degenerate primers were designed based on conserved sequences in *AAE13* cDNA from *Arabidopsis thaliana* (AAM61199, NP_190468, NP_174340 and NP_174849), *Solanum lycopersicum* (XP_010314289, XP_004234395, XP_004233163 and XP_004252541), and *Vitis vinifera* (CBI36114, XP_002267459, XP_010655748 and XP_002285808). Degenerate primers (see Supplementary Table S1 at *JXB* online) were used to generate PCR products from *Petunia* cDNA. The remaining 5ʹ and 3ʹ cDNA sequences were isolated using rapid amplification of cDNA ends (RACE). Full-length cDNAs for *PhAAE13*, *PhAAE3*, *PhACC1*, and *PhACC2*, and the partial cDNA for *PhAAE14*, were isolated by RT-PCR using the specific primers (Supplementary Table S1).

### Sequence analysis

Alignments were conducted using DNAMAN software, and a phylogenetic tree was generated using MEGA version 3.1 (Kumar *et al.*, 2004). An identity search for nucleotides and translated amino acids was conducted using the National Center for Biotechnology Information (NCBI) BLAST network server (https://blast.ncbi.nlm.nih.gov/Blast.cgi).

### Quantitative real-time PCR assays

Quantitative real-time PCR (qPCR) assays were performed according to Liu *et al.* (2011). Analyses were conducted following the Minimum Information for Publication of Quantitative Real-Time PCR Experiments guidelines (Bustin *et al.*, 2009; Tan *et al.*, 2014). Two genes, *Actin* (accession number: FN014209) and *Cyclophilin* (*CYP*) (accession number: EST883944), were used as internal reference genes to quantify the cDNA abundance (Mallona *et al.*, 2010). Similar results were obtained for both reference genes, and the data presented in this paper represent relative expression values calculated using *Actin*. The sequences of all primers used for qPCR analysis are described in Supplementary Table S2. Three biological replicates were analyzed for each treatment.

### Ethylene treatment

Petunia flowers were treated with ethylene according to previously described protocols (Spitzer-Rimon *et al.*, 2012; Tan *et al.*, 2014). Flowers were harvested at anthesis and the stems were re-cut to 5 cm, placed in flasks with distilled water, sealed, and subsequently treated with 2 µl l^−1^ ethylene for 0, 4, 8 and 16 h. The corollas from 8–10 flowers were collected at each time point, immediately frozen in liquid nitrogen, and stored at –80 °C for subsequent RNA extraction. Three biological replicates were analyzed for each treatment.

### UV-B treatment

Petunia flowers were treated with UV-B according to previously described protocols (Ryan *et al.*, 1998; Zhou *et al.*, 2007). Flowers were harvested at anthesis and the stems were re-cut to 5 cm, placed in flasks with distilled water, and subsequently exposed to UV-B at 7.2 W m^−2^ for 1, 3, and 5 h and then transferred to darkness. Control flowers were maintained for 1, 3, and 5 h in darkness. The corollas were collected at each time point, and total RNA was extracted from these samples for qPCR analysis. Three biological replicates were analyzed for each treatment.

### Agroinoculation of tobacco rattle virus vectors

To generate tobacco rattle virus (TRV) plasmids (pTRV2) containing the 3ʹ untranslated region of *PhAAE13*, *PhAAE3*, and *PhAAE14* (TRV-PhAAE13, TRV-PhAAE3, and TRV-PhAAE14), sequences of approximately 250 bp of each gene were amplified by PCR using forward and reverse primers (Supplementary Table S3), and the PCR products were inserted into the pTRV2 vector. *Agrobacterium tumefaciens* (strain GV3101) transformed with pTRV1 and pTRV2 derivatives was prepared as previously described (Spitzer-Rimon *et al.*, 2012; Tan *et al.*, 2014). The *Agrobacterium* culture was grown overnight at 28 °C in Luria-Bertani medium with 50 mg l^−1^ kanamycin and 200 mM acetosyringone. The cells were harvested and resuspended in inoculation buffer containing 10 mM 2-(*N*-morpholino)ethanesulfonic acid (MES), pH 5.5, 200 mM acetosyringone, and 10 mM MgCl_2_, to an optical density, measured at 600 nm, of 10. Following an additional 3 h of incubation at 28 °C, the bacteria containing pTRV1 were mixed with the bacteria containing the pTRV2 derivatives in a 1:1 ratio. Next, 200 to 400 ml of this mixture was applied to the cut surface of 3-week-old petunia plantlets after removal of the apical meristems. Approximately 25 plants were inoculated with each vector. The inoculated plants were grown under greenhouse conditions (22–25 °C, 14 h light/10 h dark).

### Anthocyanin extraction and analysis

Anthocyanin extraction and analysis was performed as previously described (Spitzer *et al.*, 2007).

Petunia flowers were harvested at anthesis (corollas 90° reflexed) and the corollas were collected. The stems and leaves were collected 4 weeks after infection. Three biological replicates were analyzed for each treatment.

### Malonic acid measurement

The extraction of water-soluble metabolites from petunia corollas and the subsequent analysis using GC-MS were based on a published protocol (Broeckling *et al.*, 2005; Chen *et al.*, 2011). Briefly, ~ 30 mg corollas were ground in the presence of liquid nitrogen, and subsequently 0.5 ml chloroform was added. The sample was thoroughly vortexed and incubated for 60 min at 50 °C. Next, 0.5 ml of water containing 80 mg ml^–1^ ribitol was added. The sample was incubated for an additional 60 min. After cooling to room temperature, the biphasic solvent system was centrifuged at 2900 *g* for 30 min to separate the layers. Subsequently, 0.35 ml of the polar layer was collected, transferred to a new vial, and dried in a Speed-Vac concentrator. The dried polar extracts were methoximated with 100 ml MOX reagent (2% methoxyamine HCl in pyridine; Pierce Biotechnology) at 50 °C for 30 min. The metabolites were derivatized with 100 ml *N*-methyl-*N*-(trimethylsilyl)trifluoroacetamide/1% trimethylchlorosilane (Pierce Biotechnology) for 30 min at 50 °C. The sample was subsequently transferred to a 200 ml glass insert and analyzed by GC-MS.

Next, 1.0 ml of the solution was injected at a 15:1 split ratio on to a HP 6890 GC equipped with a 30 m Rtx-5 Sil MS column (Restek; 0.25 mm internal diameter and 0.25 mm film thickness) coupled to a HP 5973 MS. The injection port and transfer arm were maintained at 280 °C. Separation was achieved with a temperature program of 80 °C for 3 min, ramped at 5 °C/min to 315 °C and maintained for 6 min. The MS source was maintained at 230 °C, and the quadrupole was maintained at 150 °C and scanned using a mass-to-charge ratio of 50–800. Three biological replicates were analyzed for each treatment.

### Fatty acid analysis

The fatty acid composition and content analysis was performed as previously described by Park *et al.* (2015) with minor modification. Approximately 10 mg of corollas were transmethylated at 95 °C for 90 min in 1 ml methanol containing 5% (v/v) H_2_SO_4_. Glyceryl triheptadecanoate was added to each sample as an internal standard. The mixture was cooled to room temperature for approximately 30 min. After transmethylation, 1.5 ml aqueous 0.9% NaCl was added, and the fatty acid methyl esters (FAMEs) were recovered by three sequential extractions with 1 ml hexane. Total FAMEs were concentrated under nitrogen gas and analyzed via gas chromatography (GC-2010; Shimadzu, Japan) on a 30 m × 0.32 mm DB-23 column (Agilent, USA). The oven temperature was held at 190 °C for 10 min and then varied from 160–230 °C at 5 °C min^−1^. The final oven temperature was maintained at 230 °C for 10 min. The fatty acids were identified based on comparison of retention times and mass spectra against standards. Three biological replicates were analyzed for each treatment.

### Cuticular wax components analysis

The cuticular wax components analysis was performed as previously described (King *et al.*, 2007). Cuticular waxes were extracted from 8–12 g of petal tissue by washing once with 100 ml and once with 50 ml hexane for 30 s. The hexane was removed from the combined extracts by rotary evaporation. The wax fraction (< 30 mg) was then dissolved in hexane and applied to a 1 g silica gel column, which had been equilibriated with hexane. Lipids were then eluted with 10 ml of (i) hexane, (ii) pentene-stabilized chloroform, (iii) acetone, and (iv) methanol, and wax-esters were eluted in the chloroform. Fractions were then analyzed by GC-MS. A Shimadzu GCMS-QP2010 Ultra was used to perform all GC-MS analyses. Sample injections volumes were 1 µl. Plant waxes were analyzed using a Rtx-5MS column (30 m, 0.25 mm internal diameter, 0.25 µm film; Restek, Bellefonte, PA, USA) using a temperature program of 100 °C for 1 min, increasing to 300 °C at 20 °C min^−1^, held at 300 °C for 10 min, increasing to 350 °C at 20 °C min^−1^, then held at 350 °C for 10 min. Three biological replicates were analyzed for each treatment.

### Statistical analyses

Statistical analysis was performed using one-way ANOVA followed by Duncan’s multiple range test with at least three replicates. Values of *P*≤0.05 were considered significant.

## Results

### Cloning the petunia *PhAAE13* cDNA sequence

Using the homologous cloning and RACE-PCR method, the full-length cDNA of *PhAAE13*, containing a 1794 bp open reading frame, was obtained. The predicted PhAAE13 protein comprises 597 amino acids and has a calculated molecular mass of 66.3 kDa.

**Fig. 2.**
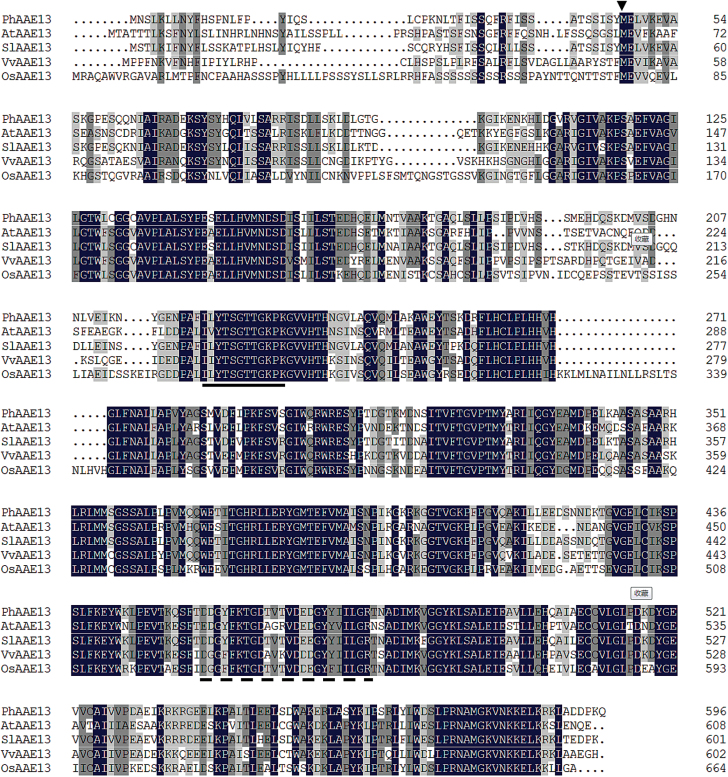
Alignment of PhAAE13 with *A. thaliana* AtAAE13 (BAB02683, AAM61199), *S. lycopersicum* SlAAE13 (XP_010314289), *V. vinifera* VvAAE13 (XP_002279139), and *O. sativa* OsAAE13 (EEC71525). White text on a black background indicates identical residues across all five sequences; dark gray shading indicates identical residues in four out of five sequences; light gray shading indicates similar residues in three out of five sequences and/or conserved substitutions. The arrow represents the start site of the sequence of another protein (ctAAE13) that is localized to the cytosol in Arabidopsis. The sequence underlined with a solid line is the conserved 12-amino acid AMP binding motif and that underline with a dotted line is the ACS (acyl-CoA synthetase) conserved domain. The alignments were generated using DNAMAN software.

The results of the multiple sequence alignment showed that PhAAE13 had 68.1% identity to AtAAE13 (accession number: AAM61199) and 87.4% identity to SlAAE13 (accession number: XP_010314289). Similar to other clade VII AAE proteins, the primary structure of predicted PhAAE13 contains conserved AMP-binding and ACS (acyl-CoA synthetase) domains (Fig. 2; Black and DiRusso, 2007; Black *et al.*, 1997; Shockey *et al.*, 2003; Weimar *et al.*, 2002). A detailed sequence alignment of the clade VII AAE homologs showed a highly conserved AMP-binding motif (PS00455) and ACS domains (Fig. 2).

### Phylogenetic analysis of AAE13 proteins

**Fig. 3.**
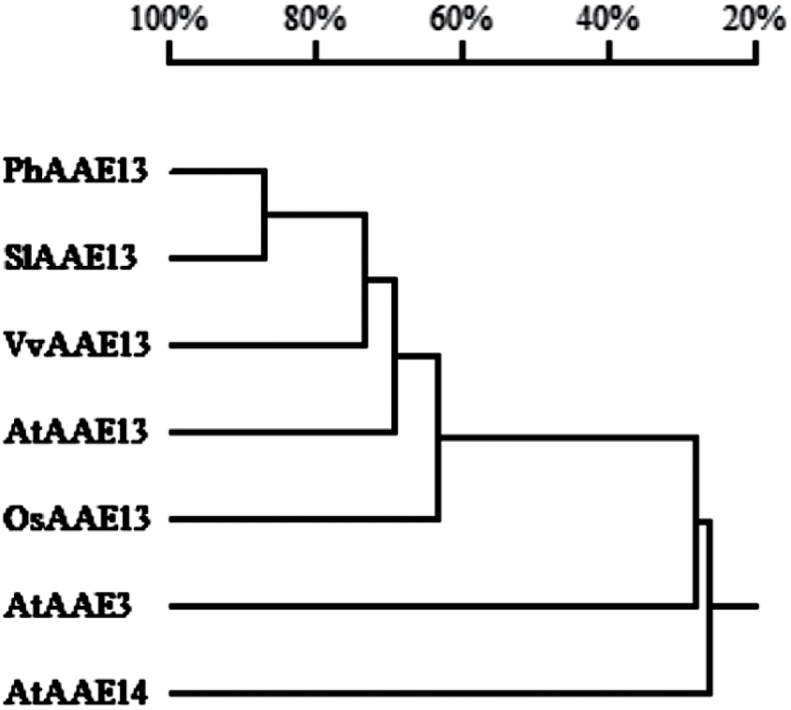
Phylogenetic tree of clade VII of AAEs. Petunia PhAAE13 was aligned with *A. thaliana* AtAAE13 (AAM61199), AtAAE3 (NP_190468), AtAAE14 (NP_174340), *S. lycopersicum* SlAAE13 (XP_010314289), *V. vinifera* VvAAE13 (CBI36114), and *O. sativa* OsAAE13 (EEC71525) using DNAMAN.

Because AAE13 belongs to clade VII of the AAE superfamily (Shockey *et al.*, 2003), we constructed a phylogenetic tree based on the AAE13 protein sequences of five plants and Arabidopsis AAE3 and AAE14, members of clade VII of the AAE superfamily. The phylogenetic tree showed that the PhAAE13 protein is most similar to AtAAE13 (Fig. 3).

To further elucidate the evolutionary relationship among AAE13-like proteins in plants, we examined the AAE13-like amino acid sequences of all known plant proteins. Using amino acid sequences derived from GenBank, a phylogenetic tree was generated with MEGA software (Supplementary Fig. S1). In all plant species examined to date, a small gene family encodes the homomeric AAE13. In most plants, one or two genes encode AAE13, except in *Brassica napus*, which has three AAE13s. The PhAAE13-coding amino acid sequence showed 57.6% and 60.9% identity to *Selaginella moellendorffii* SmAAE13a and SmAAE13b, respectively. In addition, the monocotyledon rice OsAAE13 showed 54.6% and 57.4% identity to the pteridophyte *S moellendorffii* SmAAE13a and SmAAE13b, respectively, suggesting that AAE13 protein sequences are highly conserved.

### Expression analysis of *PhAAE13*

The expression pattern of *PhAAE13* was examined in different plant organs, during bud development and in response to ethylene treatment using qPCR.

**Fig. 4.**
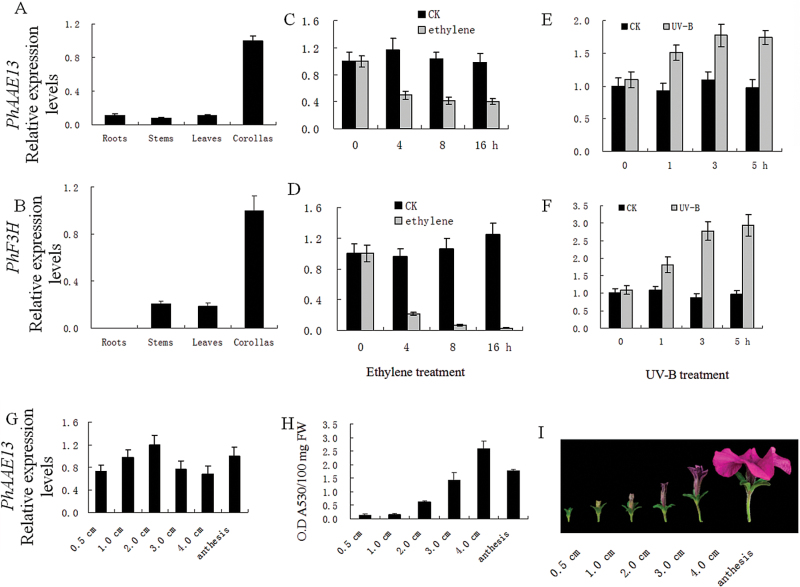
Expression of *PhAAE13* determined using qPCR. (A, B) Expression of (A) *PhAAE13* and (B) *PhF3H* in different organs. (C, D) Expression of (C) *PhAAE13* and (D) *PhF3H* in corollas in response to exogenous ethylene, (E, F) Expression of (E) *PhAAE13* and (F) *PhF3H* in corollas in response to UV-B. (G) Expression of *PhAAE13* and (H) anthocyanin accumulation in corollas during flower development. Relative expression levels are shown as fold-change values. Data are presented as means±SD (*n*=3). Three repetitions are included in the data presented. Data were generated from different flowers from different plants grown in parallel. (I) Images showing the six developmental stages of petunia buds. (This figure is available in colour at *JXB* online.)

As shown in Fig. 4A, qPCR analysis demonstrated that the mRNA levels of *PhAAE13* were highest in the corollas and lower in the roots, stems and leaves, similar to the expression of *Petunia flavonone 3-hydroxylase* (*PhF3H*) (accession number: AF022142), an anthocyanin synthesis gene (Fig. 4B).

Ethylene affects light-induced anthocyanin synthesis (Craker and Wetherbee, 1973). In the present study, the expression of *PhAAE13* and *PhF3H* in corollas significantly decreased after ethylene treatment for 4–16 h (Fig. 4C, D).

Because flavonols often accumulate to high levels in flowers, providing protection from UV-B light (Albert *et al.*, 2009; Li *et al.*, 1993; Ryan *et al.*, 2002), the effects of UV-B on *PhAAE13* expression were examined using qPCR. The transcriptional level of *PhAAE13* in corolla significantly increased from 1 to 3 h and remained stable from 3 to 5 h after UV-B treatment; similar results were seen for *PhF3H* (Fig. 4E, F).

To examine the expression of *PhAAE13* during bud development, this process was divided into six stages: S1 (0.5 cm), S2 (1.0 cm), S3 (2.0 cm), S4 (3.0 cm), S5 (4.0 cm) and S6 (anthesis) (Fig. 4I). The qPCR analysis revealed that the expression of *PhAAE13* is high during floral bud development, increases from S1 to S3, then decreases until S5, and slightly increases again at anthesis (S6) (Fig. 4G). The pigment of corollas gradually deepened, and the anthocyanin accumulation increased from stage S1 to S5 (Fig. 4G, H).

### Silencing of *PhAAE13* results in a significant reduction of anthocyanin content in petunia

To characterize *PhAAE13* functionally, a loss-of-function approach was implemented using a TRV-mediated VIGS strategy optimized for *Petunia* ‘Ultra’ (Violet line) (Tan *et al.*, 2014).

Following the known requirements for efficient gene silencing (Burch-Smith *et al.*, 2006), the construct for silencing *PhAAE13* was designed. To ensure that the dedicated VIGS construct targeted *PhAAE13*, approximately 250 bp fragments of 3ʹ untranslated sequences of the *Petunia PhAAE13* and *chalcone synthase J* (*PhCHSJ*) cDNAs were cloned from petunia cDNA into a pTRV2 vector to examine the silencing of *PhAAE13* and *PhCHS* (positive control), respectively.

**Fig. 5.**
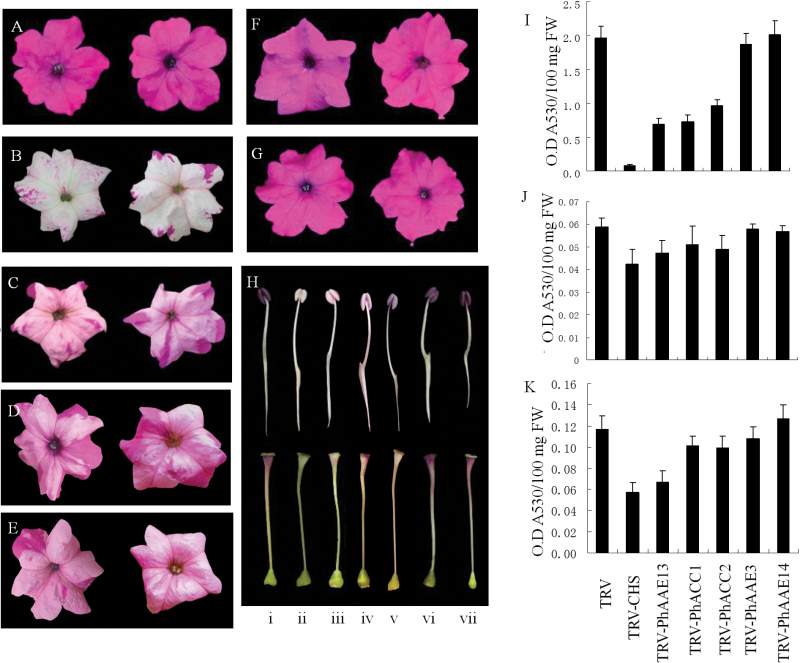
*PhAAE13* silencing reduced anthocyanin accumulation in petunia flowers. (A–G) Appearance of the corollas of (A) control (TRV-infected) and (B) *PhCHS*-, (C) *PhAAE13*-, (D) *PhACC1*-, (E) *PhACC2*-, (F) *PhAAE3*-, and (G) *PhAAE14*-silenced plants. (H) Appearance of the anthers (upper) and styles (lower) of (i) control and (ii) *PhCHS*-, (iii) *PhAAE13*-, (iv) *PhACC1*-, (v) *PhACC2*-, (vi) *PhAAE3*-, and (vii) *PhAAE14*-silenced plants. (I–K) Effects of *PhAAE13*, *PhACC1*, *PhACC2*, *PhAAE3*, and *PhAAE14* silencing on the anthocyanin content of (I) corollas, (J) leaves, and (K) stems 1 month after infection, with TRV-based *PhCHS* silencing as a positive control. Data were generated from different flowers from at least three different plants grown in parallel. Data are presented as the means±SD of three independent measurements. Statistical analysis was performed using one-way ANOVA followed by Duncan’s multiple range test with three replicates. Values of *P*≤0.05 were considered significant.

Four weeks after infection, the flowers of TRV-PhAAE13-infected and TRV-PhCHS-infected plants showed a loss of anthocyanin pigmentation phenotype to various degrees (Fig. 5B, C), but the flowers of control plants and TRV-infected plants remained purple (Fig. 5A).

In total, the corollas of some flowers in one plant infected with TRV containing the *PhAAE13* or *PhCHS* fragment were uniformly white to different degrees, and some flowers showed reduced pigmentation in the tube and at the base of the limb. However, infection with TRV containing the *PhCHS* fragment decreased anthocyanin production in the corollas to much lower levels compared with infection with TRV containing the *PhAAE13* fragment (Fig. 5B, C, I); infection with TRV containing the *PhCHS* fragment decreased anthocyanin production in the corollas by up to 95% relative to controls, while infection with TRV containing the *PhAAE13* fragment decreased anthocyanin production by up to 65% relative to controls. Moreover, anthocyanin production in the filaments and styles of the white flowers in *PhAAE13*- and *PhCHS*-silenced plants was reduced (Fig. 5H). In addition, the content of anthocyanins in mature leaves and stems was significantly reduced in *PhAAE13*-silenced plants (Fig. 5J, K), although this reduction was not visible to the human eye.

**Fig. 6.**
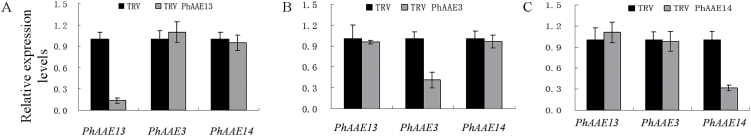
Effects of (A) TRV-PhAAE13, (B) TRV-PhAAE3, and (C) TRV-PhAAE14 treatment on the expression of *PhAAE13*, *PhAAE3*, and *PhAAE14* in flowers at anthesis as determined by qPCR. Relative expression levels are shown as fold-change values. Data are presented as means±SD (*n*=3).

Transcript accumulation was examined using primers that anneal outside the gene region of *PhAAE13* targeted for silencing. Compared with transcript accumulation in control plants, the mRNA accumulation of *PhAAE13* in white flowers of *PhAAE13*-silenced plants was significantly reduced (Fig. 6A). The expression levels of *PhAAE13* in the purple flowers of the infected plants were similar to those in the flowers of control plants.

Previous studies have shown decreased fertility in Arabidopsis *aae13* mutants (Chen *et al*, 2011). However, in the present study, fertility was not significantly affected, and fruits and seeds developed normally in *PhAAE13*-silenced plants. Other growth behaviors of *PhAAE13*-silenced plants were indistinguishable from those of control plants (Supplementary Fig. S2).

### *PhAAE13* silencing slightly increases the expression of *PhACC1* and *PhACC2*

**Fig. 7.**
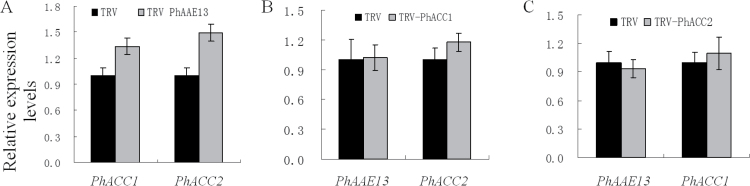
Effects of (A) TRV2-PhAAE13 treatment on the expression of *PhACC1* and *PhACC2*, (B) TRV-PhACC1 treatment on the expression of *PhAAE13* and *PhACC2*, and (C) TRV-PhACC2 treatment on the expression of *PhAAE13* and *PhACC1* in flowers at anthesis as determined using qPCR. Data are presented as means±SD (*n*=3).

It has been suggested that ACC catalyzes the biosynthesis of malonyl-CoA (Belkebir and Benhassaine-Kesri, 2013; Fatland *et al.*, 2005). Thus, we examined the effects of *PhAAE13* silencing on the expression of *PhACC*. First, we isolated two full-length *PhACC* cDNAs, named *PhACC1* and *PhACC2*, using the homology cloning method. Alignment and phylogenetic analysis confirmed that PhACC1 and PhACC2 are the homologs of Arabidopsis ACC1 and ACC2 (Supplementary Figs S3 and S4). qPCR analysis showed that the mRNA levels of both *PhACC1* and *PhACC2* were slightly but significantly increased in the corollas of *PhAAE13*-silenced plants compared with wild-type plants (Fig. 7A).

### *PhAAE13* silencing increases malonic acid accumulation in corollas

**Fig. 8.**
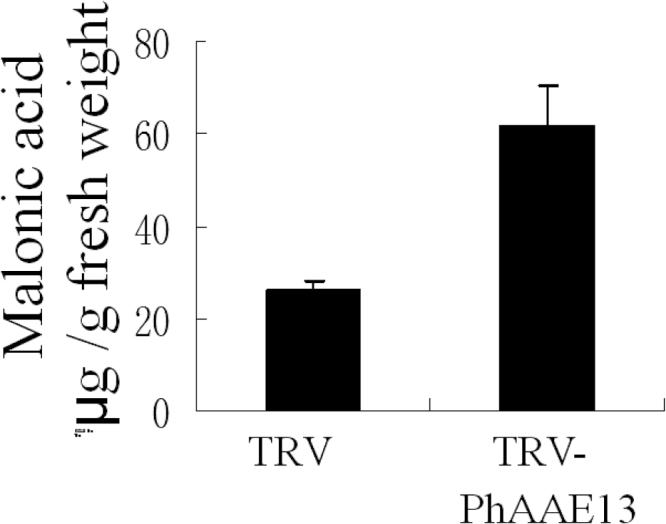
Effects of TRV2-PhAAE13 on concentrations of malonic acid in flowers at anthesis. Data are presented as means±SD (*n*=3). Statistical analysis was performed using one-way ANOVA followed by Duncan’s multiple range test with three replicates. Values of *P*≤0.05 were considered significant.

To further understand the function of PhAAE13, the malonic acid content in white corollas of *PhAAE13*-silenced plants at anthesis was examined. GC-MS was used to measure the tissue concentrations of malonic acid in extracts from white corollas at anthesis. In the flowers of *PhAAE13*-silenced plants, the malonic acid levels were 60 µg g^–1^ fresh weight, comparable to that in wild-type flowers, which contained 25 µg g^–1^ fresh weight (Fig. 8). This result showed that *PhAAE13* silencing induced the accumulation of malonic acid in corollas.

### Effects of silencing of *PhAAE13* on fatty acid contents in corollas

Because malonyl-CoA is the precursor of fatty acid biosynthesis, we examined the fatty acid content in corollas of *PhAAE13*-silenced and control plants. The content of 5 of the 25 fatty acids examined was significantly reduced in *PhAAE13*-silenced plants compared with that of control plants; among these, a reduction of 54% of the content of C22:2 (cis-13-docosenoic acid) and 37% of C23:0 (tricosanoic acid), relative to controls, was detected (Supplementary Table S5). The content of the other 20 fatty acids detected was not significantly changed in *PhAAE13*-silenced plants.

### Effects of *PhAAE13* silencing on the cuticular wax components content of corollas

Because cytosolic malonyl-CoA is also the precursor for the biosynthesis of cuticular wax components, we examined the cuticular wax components content in *PhAAE13*-silenced and control plants. The content of 6 of the 10 cuticular wax components examined was significantly reduced in *PhAAE13*-silenced plants compared with control plants; among these components, a reduction of 46.4% of the content of C25 esters and 41.5% of C23 esters was detected (Supplementary Fig. S5).

### Silencing of both *PhACC1* and *PhACC2* results in a significant reduction of anthocyanin content in petunia

We further examined whether *PhACC1* and *PhACC2* are involved in anthocyanin accumulation. TRV-PhACC1 and TRV-PhACC2 vectors were constructed and used to silence *PhACC1* and *PhACC2* in petunia. The flowers of *PhACC1*-, *PhACC2*-, and *PhAAE13*-silenced plants all showed similar anthocyanin pigmentation phenotypes and anthocyanin accumulation in flowers (Fig. 5C, D, E, H, I). However, in leaves and stems, *PhACC1*- and *PhACC2*-silenced plants did not show significant reductions in anthocyanin pigmentation compared with control plants (Fig 5J, K).

qPCR showed that the level of *PhACC1* mRNA was significantly reduced in the pink flowers of TRV-PhACC1 treated plants, while levels of *PhAAE13* and *PhACC2* were not significantly changed (Fig. 7B, C). Similarly, the level of *PhACC2* mRNA was significantly reduced in the flowers of TRV-PhACC2 treated plants, while levels of *PhAAE13* and *PhACC1* were not significantly changed.

### Silencing of the other two genes in clade VII of *PhAAE*s does not change the anthocyanin content of flowers

To further examine the specificity of *PhAAE13* involvement in anthocyanin accumulation, the cDNAs encoding *PhAAE3* and *PhAAE14*, which belong to clade VII of the *PhAAE* superfamily, were isolated. The multiple sequence alignment and phylogenetic analysis of clade VII of PhAAEs and their homologs in Arabidopsis are shown in Supplementary Figs S6 and S7.

When TRV-PhAAE3 and TRV-PhAAE14 vectors were used to silence *PhAAE3* and *PhAAE14* in petunia, the flowers of *PhAAE3*- and *PhAAE14*-silenced plants and the TRV control all showed the same anthocyanin pigmentation phenotype and anthocyanin accumulation in the flowers, leaves and stems (Fig 5A, F, G, H, I). In addition, the colors of the filaments and styles of the flowers of *PhAAE3*- and *PhAAE14*-silenced plants were not significantly changed compared with the TRV control (Fig. 5H). The qPCR analysis showed that the expression of *PhAAE3* and *PhAAE14* was significantly reduced in plants infected with the corresponding (TRV-PhAAE3 and TRV-PhAAE14) vectors, while the expression of *PhAAE13*, *PhAAE3*, and *PhAAE14* was not significantly changed in plants infected with vectors carrying the homologous genes (Fig. 6A).

## Discussion

Anthocyanins are the major pigments in the flowers of higher plants. Many structural genes of anthocyanin biosynthesis, including *CHI*, *CHS*, *F3H*, and *DFR*, have been cloned and identified (Weiss, 2000). Malonyl-CoA is an important intermediate in anthocyanin synthesis (Mol *et al.*, 1996). Previously, ACC was identified as the key enzyme of malonyl-CoA synthesis in anthocyanin biosynthesis (Sasaki and Nagano, 2004). Recently, AAE13 was identified as malonyl-CoA synthetase in Arabidopsis (Chen *et al.*, 2011). The results obtained in the present study show that PhAAE13, the key synthase of malonyl-CoA, plays an important role in anthocyanin biosynthesis in petunia flowers.

In the present study, we cloned the full-length cDNA of *PhAAE13*. The phylogenetic tree (Fig. 3) showed that PhAAE13 protein was most similar to AtAAE13 in Arabidopsis, suggesting that PhAAE13 is a homolog of AtAAE13. The phylogenetic tree of the AAE13s in all known plants in NCBI and the high similarity between the AAE13 members demonstrated that AAE13 is a small, conserved gene subfamily (see Supplementary Fig. S1).

Flavonoid biosynthesis genes are typically coordinately regulated by developmental and environmental cues, such as growth stage (Lepiniec *et al.*, 2006). Furthermore, metabolic channeling has been proposed for flavonoid biosynthesis enzymes (Jørgensen *et al.*, 2005; Winkel, 2004), suggesting the need for tight co-regulation of protein amounts. In the petunia ‘Ultra’, the anthocyanins primarily accumulated in the flower organs. The expression of *PhAAE13*, similar to that of *PhF3H*, was highest in corollas. Flavonoid accumulation in seedlings is developmentally regulated and parallels the expression of the early genes of flavonoid biosynthesis, *CHS*, *CHI*, and *F3H* in *Arabidopsis* (Pelletier *et al.*, 1999). Here, we observed that the accumulation of anthocyanins in buds is developmentally regulated, and, although there is no close correlation between the anthocyanin content and *PhAAE13* expression during development of the corollas, *PhAAE13* expression is not low (Fig. 4G, H). A previous study has identified two types of AAE13 transcripts, with the resulting proteins being localized to the cytosol and mitochondria, respectively (Guan and Nikolau, 2016); this observation could partially explain the lack of close correlation between the anthocyanin content and *PhAAE13* expression during development of the corollas.

Ethylene markedly suppresses anthocyanin accumulation (Craker and Wetherbee, 1973), while the Co^2+^-mediated inhibition of ethylene biosynthesis and the prevention of ethylene activity by silver increases the anthocyanin content of maize (*Zea mays*) seedlings (Rengel and Kordan, 1987). Similarly, the petals of transgenic tobacco (*Nicotiana tabacum*) plants expressing the mutant melon (*Cucumis melo*) ethylene receptor gene *ethylene response1* (ETR1H69A) accumulate higher levels of anthocyanins than control plants (Takada *et al.*, 2005). In the present study, ethylene treatment decreased the expression of *F3H*, an anthocyanin biosynthesis gene, suggesting the negative regulation of ethylene on anthocyanin biosynthesis in petunia corollas; the level of *PhAAE13* mRNA was also down-regulated by ethylene (Fig. 4C, D). Flavonoids are strong UV-absorbing metabolites that primarily accumulate in epidermal cells after UV induction, suggesting that these molecules function as a protective shield (Schmelzer *et al.*, 1988). One of the most general responses of plants to UV light is the transcriptional activation of flavonoid biosynthesis genes (Abbruzzese *et al.*, 1986; Favory *et al.*, 2009; Koes *et al.*, 1994; Ryan *et al.*, 2002). Consistently, in the present study, the mRNA levels of both *PhAAE13* and *PhF3H* were up-regulated after UV-B treatment (Fig. 4E, F).

Chen *et al.* (2011) identified AtAAE13 as malonyl-CoA synthase, which catalyzes the formation of malonyl-CoA from malonic acid. Malonyl-CoA is the precursor for the formation of flavonoids and anthocyanins (Peer *et al.*, 2001). In the present study, *PhAAE13* silencing reduced anthocyanin biosynthesis and malonic acid accumulation. These results further suggested that the formation of malonyl-CoA is catalyzed through AAE13, with malonic acid as the substrate. The canonical view of flavonoid and anthocyanin biosynthesis suggests that malonyl-CoA is almost exclusively formed via acetyl-CoA carboxylase, which catalyzes the ATP-dependent formation of malonyl-CoA from acetyl-CoA and bicarbonate. In the present study, *PhAAE13* silencing increased malonic acid accumulation and significantly reduced the anthocyanin content of corollas (Figs 5C and 8), suggesting that PhAAE13 is an alternative enzymatic source of precursors for anthocyanin biosynthesis in petunia flowers. In addition, the amounts of 5 of the 25 fatty acids and 6 of the 25 cuticular wax components detected were significantly reduced in *PhAAE13*-silenced plants compared with controls, suggesting a role for PhAAE13 in the biosynthesis of fatty acids and cuticular wax components in petunia.

CHS is a unique enzyme that catalyzes the synthesis of chalcone by coumaroyl-CoA. In the present study, VIGS-mediated silencing of *PhCHS* showed a stronger reduction of anthocyanin biosynthesis than that associated with *PhAAE13* silencing. In addition, there was the patchy appearance typical of VIGS. Infection with TRV-PhCHS led to white patches, while infection with TRV-PhAAE13, TRV-PhACC1, and TRV-PhACC2 led to pink patches (Fig. 5C), which was repeatedly observed. These results indicate that in addition to PhAAE13, malonyl-CoA synthesis is catalyzed by another enzyme, PhACC1 and PhACC2 in petunia flower.

In a study in Arabidopsis, all of the homozygous *aae13* mutant plants exhibited strong defects in growth and development, and after 39 d growth, many of the mutant plants died and the others remained small and chlorotic (Chen *et al.*, 2011). However, in the present study, except for the reduction of anthocyanin biosynthesis in the flowers, the growth behavior of *PhAAE13*-silenced plants was indistinguishable from that of control plants. It is likely that infection with TRV-PhAAE13 led to partial *PhAAE13* silencing in petunia (Fig. 6A), while homozygous *aae13* mutant plants show loss-of-function mutations. Moreover, in Arabidopsis, all wild-type and hemizygous *aae13* plants showed normal growth and development (Chen *et al.*, 2011). In addition, the height of the seedlings after infection was 10–15 cm, and the effects of *PhAAE13* silencing on young seedlings were not observed.

*PhAAE13* belongs to clade VII of the *AAE* superfamily, which in Arabidopsis contains three genes, *AtAAE13*, *AtAAE3*, and *AtAAE14*. Kim *et al.* (2008) and Foster *et al.* (2012) identified Arabidopsis AtAAE14 (At1g30520) and AAE3 (At3g48990) as *o*-succinylbenzoyl-coenzyme A ligases acting in phylloquinone and oxalyl-CoA synthetase, respectively; whether AtAAE14 and AtAAE3 are involved in anthocyanin biosynthesis remains unknown. In the present study, petunia *PhAAE3* and *PhAAE14* were isolated, and VIGS-mediated silencing of both *PhAAE3* and *PhAAE14* did not change anthocyanin biosynthesis in petunia. The genes from clade VII almost defy categorization into a single clade because all three sequences are quite divergent relative to the other members of the superfamily (Shockey *et al.*, 2003). These results suggested that among the members of clade VII of the *AAE* superfamily, *PhAAE13* is specifically involved in anthocyanin biosynthesis. In addition, the increase in the level of *PhACC* mRNA could reflect a compensation mechanism of malonyl-CoA biosynthesis in *PhAAE13*-silenced corollas. These findings may partially explain that PhAAE13 could be redundant or predominant over cytosolic ACC isoforms.

Recently, Guan and Nikolau (2016) showed that the cytosolic AAE13 protein in Arabidopsis is not essential because there is a redundant malonyl-CoA generating system provided by a cytosolic acetyl-CoA carboxylase, while the mitochondrial AAE13 protein is essential for plant growth. The *aae13-1* mutant showed typical metabolic phenotypes associated with a deficiency in the mitochondrial fatty acid synthase system, including the depletion of lipoylation of the H subunit of the photorespiratory enzyme glycine decarboxylase, increased accumulation of glycine and glycolate, and reduced sucrose levels (Guan and Nikolau, 2016). However, in the present study, VIGS-mediated *PhAAE13* silencing reduced the production of anthocyanin, which is synthesized in the cytoplasm, suggesting that the cytosolic PhAAE13 protein is essential for anthocyanin biosynthesis in petunia corollas. In addition, the transcriptional level of the two possible types of *PhAAE13* transcripts should be reduced in plants infected with TRV-PhAAE13, which contains the common 3ʹ UTR sequence of *PhAAE13* transcripts, although the plants did not show a visible change in plant growth; the cause of this finding could be that the VIGS-mediated approach achieved only partial silencing of *PhAAE13*.

## Conflict of interest

The authors declare that there are no conflicts of interest.

## Author contributions

YY and LJ designed the research; CG, LH, ZH, and WQ performed the research; ZH and WQ analyzed the data; and YY and LJ wrote the paper.

## Supplementary Material

Supplementary Data
