# Supplementary material for: The acyl-activating enzyme PhAAE13 is an alternative enzymatic source of precursors for anthocyanin biosynthesis in petunia flowers
Source: J Exp Bot. 2016 Dec 7;68(3):457–67. doi: 10.1093/jxb/erw426 (PMC5441920; doi:10.1093/jxb/erw426)
Supplement: Supplementary Data [file erw426_Supplementary_Data.zip › supplementary_tables_S1_S5_figures_S1_S7.pdf]

**Supplementary Table S1** Primer sequences of *PhAAE13*, *PhAAE3*, and *PhAAE14* used in for cloning genes

| Gene | Forward primer (5′→3′) | Reverse primer (5′→3′) | Type |
|---|---|---|---|
| *PhAAE13* | TAYACNWSNGGNACNACNGGNAA | TTRTTNACYTTNCCCATNGCRTT3 | Degenerate primers |
| *PhAAE13* | AGGAGGTACTGTTGGCAAGCCA | AGCTAACATTTGGACCTGTGCT | Specific primers |
| *PhAAE3* | AARCCNGGNGAYGTNGTNGC | GGNGCNCCRAANGCYTCYTC | Degenerate primers |
| *PhAAE3* | TTGCGCTGTACCATGTGACGAC | ATTAACAACAGTTTGGATTCAG | Specific primers |
| *PhAAE14* | GCNTAYGGNATGACNGARGCNTG | GGRTANACRTTYTCNCCNCC | Degenerate primers |
| *PhAAE14* | CTCGGATACAGGAGTGAAATGA | GCCTCGGTCATTCCATAAGCTG | Specific primers |
| *PhACC* | GGNGGNGGNATHCAYGARTT | RTCRTARCARTANGTNGTRTT | Degenerate primers |
| *PhACC1* | GCGCTGTTCAGAAAATCTTGAAAG | CAATTCTACTGTCCAACCAACC | Specific primers |
| *PhACC2* | GGATAGTTGCAGTTGAGACCCA | GCTCTAACCCTCATAGCAATTCT | Specific primers |

**Supplementary Table S2** Primer sequences of *PhAAE13*, *PhAAE3*, *PhAAE14*, *PhF3H*, *PhACC1*, *PhACC2*, *PhACC*,*PhACTIN* and *PhCYF* used in quantitative real-time PCR

| Gene | Forward primer (5′→3′) | Reverse primer (5′→3′) |
|---|---|---|
| *PhAAE13* | TAGTTGTGCCTGATGCTG | CGAATCCCACAGATACAGA |
| *PhAAE3* | AACAGCTCAAGTGCCCAAAT | GTGAACCTGCAATACTCCTT |
| *PhAAE14* | TGTTTGAGCTGCTAACTACAT | TAACGGGAAATCATCAATAC |
| *PhF3H* | TAATGGATGAGCCCATAACA | CCTTCTCAGCAGCAACTTCA |
| *PhACC1* | TTGAGCCAGCAACCAGAG | CAGGAGCAAATAGCAGACAATA |
| *PhACC2* | AACTATGACGAAAGGCTAC | ACAAGTCCCTGAGGTAGA |
| *PhACTIN* | TGCTGATCGAATGAGCAAGGAA | GGAGCAACAACCTTAATCTTC |
| *PhCYP* | AGGCTCATCATTCCACCGTGT | TCATCTGCGAACTTAGCACCG |

**Supplementary Table S3** Primer sequences of *PhAAE13*, *PhAAE3*, *PhAAE14,* and *PhCHS* used in VIGS

| Gene | Forward primer (5′→3′) | Reverse primer (5′→3′) |
|---|---|---|
| *PhAAE13* | CTGGATCC AGCAAGTTTAGAACGGTCAG | CGGAATTCCAAAACAGCGCCTTTCACGT |
| *PhAAE3* | CTGGATCCGTGCCCAAATTCGGAGCTTGA | CGGAATTCGACATCATACCTAAAGACCAG |
| *PhAAE14* | CTGGATCCCTGTAGAGCGAAAGGTTTGA | CGGAATTCTGGACAATTTGTTGTAGGGA |
| *PhCHS* | GATCTCGAGTGGAGGCATTCCAACCATTG | CCAGAGCTCATTCAAGACCTTCACCAG |

**Supplementary Table S4** Comparative analysis of the amino acid sequence of PhAAE13 with their closest homologs in *Arabidopsis*, tomato, *Vitis vinifera* and *Oryza sativa*.

| Identity (%) | PhAAE13 | AtAAE13 | SlAAE13 | VvAAE13 | OsAAE13 | AtAAE14 |
|---|---|---|---|---|---|---|
| AtAAE13 | 68.1% | 100% | | | | |
| SlAAE13 | 87.4% | 68.3% | 100% | | | |
| VvAAE13 | 73.2% | 70.8% | 73.5% | 100% | | |
| OsAAE13 | 60.2% | 63.4% | 60.6% | 67.5% | 100% | |
| AtAAE3 | 28.5% | 28.3% | 28.1% | 27.7% | 29.1% | 100% |
| AtAAE14 | 25.2% | 26.1% | 25.0% | 25.6% | 24.7% | 27.1% |

**Supplementary Table S5** Effects of *PhAAE13* silencing on the content of fatty acids in corollas in *Petunia*

| | TRV (μg/g) | TRV-PhAAE13 (μg /g) | TRV-PhAAE13/TRV ratio (%) |
|---|---|---|---|
| C10:0（Decanoic acid） | 1.46±0.98a | 1.59±0.45a | 108.90 |
| C11:0（Undecanoic aci） | 11.63±1.27a | 9.12±2.66a | 78.42 |
| C12:0（Laurel acid） | 5.91±1.63a | 6.36±0.14a | 107.61 |
| C14:0（Myristic acid） | 23.94±4.98a | 25.61±6.29a | 106.98 |
| C14:1（Myristolenic acid） | 54.69±6.68a | 55.23±4.04a | 100.99 |
| C15:0（Pentadecanoic acid） | 56.39±7.10a | 52.16±2.46a | 92.50 |
| C15:1（cis-10-Pentadecenoic acid） | 32.56±7.68a | 34.86±43.37a | 107.06 |
| C16:0（Palmitic acid） | 2296.46±169.71a | 2234.59±155.87a | 97.31 |
| C16:1（Palmitoleic acid） | 4.67±0.11a | 4.19±2.12a | 89.72 |
| C17:0（Margaric acid） | 18.67±3.85a | 19.42±2.21a | 104.02 |
| C17:1（10-Heptadecenoic acid） | 5.97±0.14a | 5.43±1.06a | 90.95 |
| C18:0（Stearic acid） | 1439.46±33.71a | 1391.52±52.69a | 96.67 |
| C18:1N9T（Elaidic acid ） | 2.54±0.71a | 2.93±1.22a | 115.35 |
| C18:2N6C（Linoleic acid） | 858.47±93.64a | 884.63±68.79a | 103.05 |
| C18:2N6T（Trans-linoleic acid） | 13.72±1.00a | 11.46±3.99a | 83.53 |
| C18:3N3（α-Linolenic acid） | 446.56±13.68a | 426.53±28.74a | 95.51 |
| C18:3N6（γ-Linolenic acid） | 11.04±0.30a | 7.19±2.46a | 65.13 |
| C20:0（Arachidic acid） | 149.56±9.49a | 131.64±13.83a | 88.02 |
| **C20:1（cis-11-Eicosenoic acid）** | 103.16±2.11a | 95.49±3.86b | 92.56 |
| **C21:0（Heneicosanoic acid）** | 10.55±1.30a | 7.23±0.82b | 68.53 |
| C22:0（Behenic acid） | 341.25±21.44a | 331.11±1.81a | 97.03 |
| C22:1N9（Erucic acid） | 29.21±7.72a | 26.77±7.28a | 91.65 |
| **C22:2（cis-13-Docosenoic acid）** | 12.44±4.58a | 6.98±2.19b | 56.11 |
| **C23:0（Tricosanoic acid）** | 8.11±0.23a | 5.12±0.35b | 63.13 |
| **C24:0（Tetracosanoic acid）** | 363.79±21.47a | 311.67±7.33b | 85.67 |
| Total | 6300.75±395.42 | 6087.24±429.73 | 108.90 |

*Data are presented as the means ± SD (n = 3). Different letters indicate significant difference at P=0.05 levels. Bold font indicates significant difference. Statistical analysis was performed using one way analysis of variance (ANOVA) followed by Duncan's multiple range test (DMRT) with 3 replicates. *P*-values ≤0.05 were considered as significant.

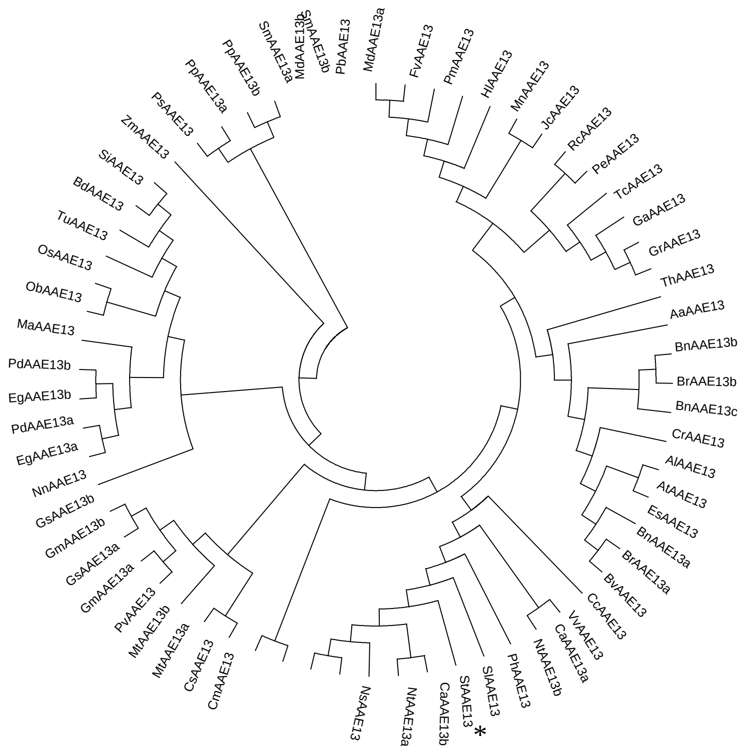


**Supplementary Figure S1** Phylogenetic tree of AAE13s. Petunia PhAAE13 (asterisk) was aligned with *Arabidopsis lyrata* AlAAE13 (XP_002882983), *Arabidopsis thaliana* AtAAE13 (AAM61199), *Arabis alpina* AaAAE13 (KFK38968), *Beta vulgaris* BvAAE13 (XP_010691378), *Brachypodium distachyon* BdAAE13 (XP_003567137), *Brassica napus* BnAAE13b (CDX97727), *Brassica napus* BnAAE13a(CDY28376), *Brassica napus* BnAAE13c (CDY30140), *Brassica rapa* BrAAE13a (XP_009114853), *Brassica rapa* BrAAE13b (XP_009146164), *Capsella rubella* CrAAE13 (XP_006297255), *Coffea canephora* CcAAE13 (CDO98014), *Cucumis melo* CmAAE13 (XP_008452305), *Cucumis sativus* CsAAE13 (XP_004149301), *Elaeis guineensis* EgAAE13a (XP_010920655), *Elaeis guineensis* EgAAE13b (XP_010924575), *Eutrema salsugineum* EsAAE13 (XP_006406869), *Fragaria vesca* FvAAE13 (XP_004302888), *Glycine max* GmAAE13a (XP_003531671), *Glycine max* GmAAE13b (XP_006583069), *Glycine soja* GsAAE13b (KHN04218), *Glycine soja* GsAAE13a (KHN47830), *Gossypium arboreum* GaAAE13 (KHG14495), *Gossypium raimondii* GrAAE13 (KJB70446), *Humulus lupulus* HlAAE13 (AGA17925), *Jatropha curcas* JcAAE13 (XP_012073388), *Malus domestica* MdAAE13a (XP_008342666), *Malus domestica* MdAAE13b (XP_008358093), *Medicago truncatula* MtAAE13a (KEH28754), *Medicago truncatula* MtAAE13b (KEH28799), *Morus notabilis* MnAAE13 (XP_010098090), *Musa acuminata* MaAAE13 (XP_009389393), *Nelumbo nucifera* NnAAE13 (XP_010254053), *Nicotiana tomentosiformis* NtAAE13a (XP_009593689), *Nicotiana sylvestris* NsAAE13 (XP_009786717), *Nicotiana tomentosiformis* NtAAE13b (XP_009628041), *Oryza brachyantha* ObAAE13 (XP_006644745), *Oryza sativa* OsAAE13 (EEC71525), *Phaseolus vulgaris* PvAAE13 (XP_007135668), *Phoenix dactylifera* PdAAE13a (XP_008789317), *Phoenix dactylifera* PdAAE13b (XP_008793069), *Physcomitrella patens* PpAAE13a (XP_001759556), *Physcomitrella patens* PpAAE13b (XP_001774260), *Picea*

*sitchensis* PsAAE13 (ABR16405), *Populus euphratica* PeAAE13 (XP_011008326), *Prunus mume* PmAAE13 (XP_008228486), *Pyrus bretschneideri* PbAAE13 (XP_009353599), *Ricinus communis* RcAAE13 (XP_002516015), *Selaginella moellendorffii* SmAAE13a (XP_002979361), *Selaginella moellendorffii* SmAAE13b (XP_002990335), *Setaria italica* SiAAE13 (XP_004970043), *Solanum lycopersicum* SlAAE13 (XP_010314289), *Solanum tuberosum* StAAE13 (XP_006359460), *Tarenaya hassleriana* ThAAE13 (XP_010545693), *Theobroma cacao* TcAAE13 (XP_007024201), *Triticum urartu* TuAAE13 (EMS65189), *Vitis vinifera* VvAAE13 (CBI36114), *Zea mays* ZmAAE13 (DAA57520), *Capsicum annuum* CaAAE13a (Capana12g001207), CaAAE13b (Capana03g000294). The amino acid sequences of AAEs were obtained from the National Center for Biotechnology Information database and The Pepper Genome database (http://peppersequence.genomics.cn/page/species/index.jsp). The amino acid sequences were analyzed with Vector NTI (version 9.0.0; Invitrogen), and the phylogenetic tree was constructed with MEGA (version 3.1) using a bootstrap test of phylogeny with minimum evolution test and default parameters.

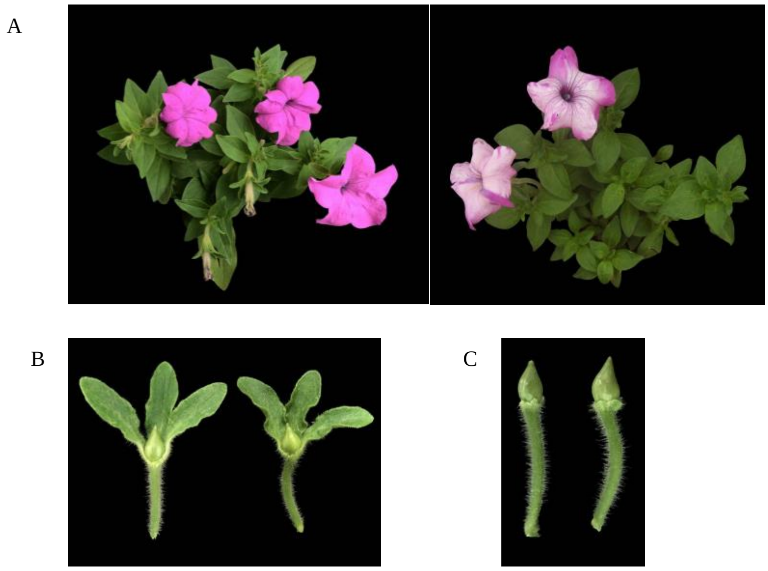


**Supplementary Figure S2** Phenotype of wild type and *PhAAE13*-silenced plants. A, Seven-week-old *PhAAE13*-silenced (right) and wild type (left) plants. B and C, fruits at three weeks after the pollination of *PhAAE13*-silenced (right) and wild type (left) plants.

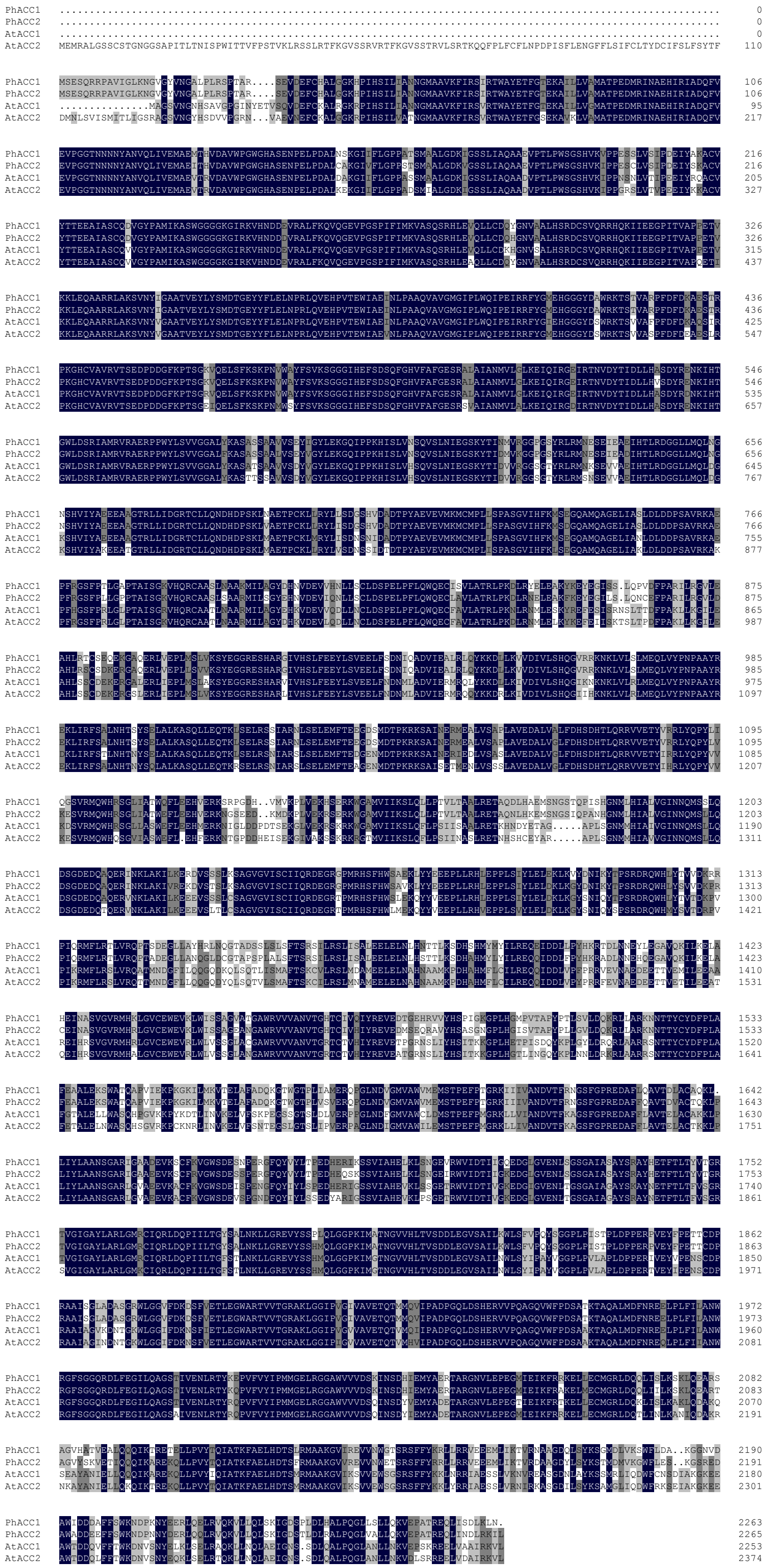

**Supplementary Figure S3** Alignment of PhACC1 and PhACC2 with *Arabidopsis thaliana* AtACC1 (AAF18638) and AtACC2 (AAG40563). Dark-gray shading indicates identical residues. The alignments were generated using DNAMAN software. Identical and similar amino acids are reversed and shaded, respectively.

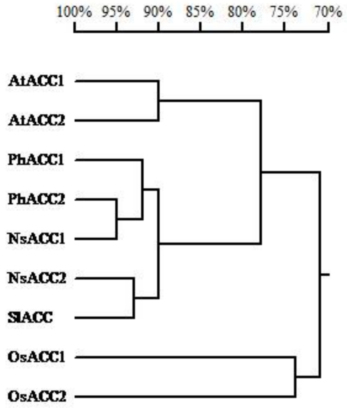


**Supplementary Figure S4** Phylogenetic analysis of ACCs. Petunia PhACC1 and PhACC2 were aligned with *Arabidopsis thaliana* AtACC1 (AAF18638), AtACC2 (AAG40563), *Nicotiana sylvestris* NsACC1 (XP_009758450), NSACC2 (XP_009799610), *Solanum lycopersicum* SlACC (XP_004252541), *Oryza sativa* OsACC1 (Q8S6N5), and OsACC1 (B9FK36), using DNAMAN.

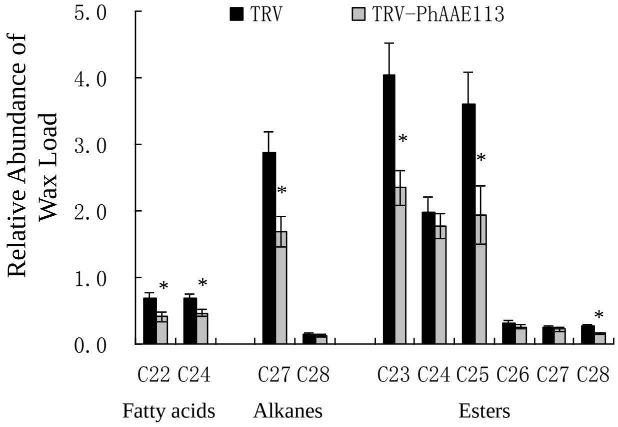


**Supplementary Figure S5** Effects of *PhAAE13* silencing on the content of cuticular wax components. Corollas were chloroform extracted and analyzed for wax using GC-FID/MS. Each wax constituent is designated by carbon chain length and is labeled by chemical class along the x-axis. Statistical analysis was performed using one way analysis of variance (ANOVA) followed by Duncan's multiple range test (DMRT) with 3 replicates. *Means significantly different ($P < 0.05$) from wild type. Error bars = SE.

```
PhAAE13    ........MNSLKLLNYFHSPNLFP....YIQS..LCPKNLTFISSQFRFISSATSSIS................YMELVKEVASKGPES     60
AtAAE13    ..............................................................................MEVFKAAFSEASNS     14
SlAAE13    ........MSTLKIFNYFLSSKATPLHSLYIQYHFSCQRYHSFISSQLRLLSSATSSIS................YMELVKEVASKGPES     66
VvAAE13    ..............................................................................MEVIKAVARQGSAT     14
OsAAE13    MRAQAWVRGAVARLMTPFNCPAAHASSSPYHLLLLPSSSYSLLSRLRRHFASSSSSSSSSRSSSPAYNTTQNTTSTFMEVVQEVLKHGSTQ     91
PhAAE3     ................................................................................MASMTLTGYLK     11
AtAAE3     ................................................................................MDSDTLSGLLE     11
PhAAE14    ...........................................................................................      0
AtAAE14    ..............................................................................MANHSRPHICQCL     13

PhAAE13    QQNIAIRADEKSYSYHQLVLSARRISDLLSKLDL.........GTGKGIKENKH.....LDGVRVGIVAKPSAEFVAGILGTWLCGGCAVP    137
AtAAE13    CDRIAIKADGKSYSYGQLTSSALRISKLFLKDDT.........TNG.GQETKKYEGFGSLKGARIGIVAKPSAEFVAGVLGTWFSGGVAVP     95
SlAAE13    QKNIAIRADEKSYSYLQLISSARRISSLLSKLDL.........KTDKGIKENEH.....HKGARVGIVSKPSAEFVAGILGTWLSGGCAVP    143
VvAAE13    AESVAIRANQKSYSYNQLISSARKISSLLCNGDI.........KPTYGVSKHKHSGNGHLGGARIGIVAKPSVEFVAGILGTWFSGGVAVP     96
OsAAE13    GVRAAIRSDQKSYNLVQLIASALDVYNILCNKNVPPLSFSMTQNGSTGSSVKGINGTGFLGGARIGIVAKPSPEFVAGIFGTWLSGGVAVP    182
PhAAE3     NVAAKFPTRRAIAVSGKFDVTHARLDELIDQAAS.......QLVG.AGVKP..........GDVVALTFANTIEFVIMFLAVIRSRATAAP     84
AtAAE3     NVAKKFPDRRALSVSGKFNLTHARLHDLIERAAS.......RLVSDAGIKP..........GDVVALTFPNTVEFVIMFLAVIRARATAAP     85
PhAAE14    ...........................................................................................      0
AtAAE14    TRLASVKRNAVVTVYGNRKRTGREFVDGVLSLAAG........LIRLGLRN..........GDVVSIAAFNSDLFLEWLLAVALVGGVVAP     86

PhAAE13    LALSYPESELLHVMNDSDISIILSTEDHQELMNTVAAKTGAQLSLLPSIPDVHS...SMEHDQSKDMVSDGHNNLVEIKN....YGENPAF    221
AtAAE13    LALSYPEAELLHVMNDSDISLLLSTEDHSETMKTIAAKSGARFHLIPPVVNSTSETVACNQFQDDSFEAEGK............FLDDPAL    174
SlAAE13    LALSYPESELLHVMNDSDISIILSTEDHQELMNAIAAKTGAQLSLIPSIPDVHS...STKHDQSKDMVSDGQQDLLEINS....YGENPAF    227
VvAAE13    LALSYPEAELLHVMNDSDVSMILSTEDYRELMENVAAKSSAQFSLIPPVPSIPSPTSARDHPQTGEIVADKS....LQGE....IDEDPAL    179
OsAAE13    LALSYPEAELLHVMNDSDISLILSTKEHQDIMENISTKCSAHCSLLPSVTSIPVN.IDCQEPSSTEVTSSISSLIAEIDSSKEIRGDDPAL    272
PhAAE3     LNAAYTSEEFEFYLSDSESKLLLIAKEGNEAAQAAATKLNIP.QITVTLPESDS..FISLTPSSPLSDSDSVSKIVN.......EPSDVAL    165
AtAAE3     LNAAYTAEEFEFYLSDSDSKLLLTSKEGNAPAQEAASKLKIS.HVTATLLDAGS..DLVLSVADSDSVVDSATELVN.......HPDDGAL    166
PhAAE14    ...........................................................................................      0
AtAAE14    LNYRWSLKEAKMAMLLVEPVLLVTDETCVSWCIDVQNGDIPSLKWRVLMESTSTDFANELNQFLTTEMLKQRTLVPSLAT.YAWASDDAVV    176

PhAAE13    ILYTSGTTGKPKGVVHTHNGVLAQVQMLAKAWEYTSKDRFLHCLPLHHVH..............................GLFNALLAPV    281
AtAAE13    IVYTSGTTGKPKGVVHTHNSINSQVRMLTEAWEYTSADHFLHCLPLHHVH..............................GLFNALFAPL    234
SlAAE13    ILYTSGTTGKPKGVVHTHNGVLAQVQMLANAWEYTPKDQFLHCLPLHHVH..............................GLFNALLAPL    287
VvAAE13    IIYTSGTTGKPKGVVHTHKSINSQVQILTEAWGYTSADQFLHCLPLHHIHKTIFRILKISISHNFDMKFLHGSLTLTIHVHGLFNALLAPL    270
OsAAE13    ILYTSGTTGKPKGVVHTHKGIVSQVQILSEAWGYRSEDQFLHCLPLHHIHKKLMLNAILN.........LLRSLTSNLHVHGLFNALFAPL    354
PhAAE3     FLHTSGTTSRPKGVPLTQHNLVSSVNNIKSVYKLTESDSTVIVCPLFHVH..............................GLIAGLLSSL    225
AtAAE3     FLHTSGTTSRPKGVPLTQLNLASSVKNIKAVYKLTESDSTVIVLPLFHVH..............................GLLAGLLSSL    226
PhAAE14    ...........................................................................................      0
AtAAE14    ICFTSGTTGRPKGVTISHLAFITQSLAKIAIAGYGEDDVYLHTSPLVHIG...............................GLSSAMAML    235

PhAAE13    YAGSMVDFIP..KFSVSGIWQRWRESYPTDGTKMDNSITVFTGVPTMYARLIQGYEAMDPELKAASASAARHLRLMMSGSSALPLPVMQQW    370
AtAAE13    YARSLVEFLP..KFSVSGIWRRWRESYPVNDEKTNDSITVFTGVPTMYTRLIQGYEAMDKEMQDSSAFAARKLRLMMSGSSALPRPVMHQW    323
SlAAE13    YAGSTVDFVP..KFSVRGIWQRWRESYPTDGTITDNAITVFTGVPTMYARLIQGYEAMDPELKTASASAARHLRLMMSGSSALPLPVMQQW    376
VvAAE13    YAGSTVEFMP..KFSVRGIWQRWRESHPKDGTKVDDAITVFTGVPTMYTRLIQGYEAMDPELQAASASAASKLRLMMCGSSALPYPVMQQW    359
OsAAE13    YSGSVVEFMP..KFSVRGIWQRWRESYPNNGSKNDEAITVFTGVPTMYTRLLQGYDGMDPEQQSASSFAAKQLRLMMCGSSALPSPLMKRW    443
PhAAE3     GAGGSVTLPASGRFSATSFWSDMKSYN..........ATWYTAVPTIHQILLDRHLSN.PE......SSYPKLRFIRSCSASLAPSILARL    299
AtAAE3     GAGAAVTLPAAGRFSATTFWPDMKKYN..........ATWYTAVPTIHQIILDRHASH.PE......TEYPKLRFIRSCSASLAPVILSRL    300
PhAAE14    ................................................DSTFTCHRTKHRS......VGSKFVKKVLNGAGSLSAELVKNA     37
AtAAE14    MVGACHVLLP..KFDAKTALQVMEQNH..........ITCFITVPAMMADLIRVNRTTKNG......AENRGVRKILNGGGSLSSELLKEA    308

PhAAE13    ETITG.HRLLERYGMTEFVMAISN.....PIKGK...............RKGGTVGKPFPGVQAKILLEEDSNNDKTGVGELCIKSPSLFK    440
AtAAE13    ESITG.HRLLERYGMTEFVMAMSN.....PLRGA...............RNAGTVGKPLPGVEAKIKEDE...NDANGVGEICVKSPSLFK    390
SlAAE13    ETITG.HRLLERYGMTEFVMAISN.....PINGK...............RKGGTVGKPFPGVQAKILLDDASSNDQTGVGELCIKSPSLFK    446
VvAAE13    ETITG.HRLLERYGMTEFVMAISN.....PLKGV...............RKGGTVGKPFPGVQVKILADE.SETETTGVGELCIKSPSLFK    428
OsAAE13    EEVTG.HRLLERYGMTEFVMALSS.....PLHGA...............RKEGTVGKPLPRVEAKIIMEDG.AETTSEVGELCIRSPSLFK    512
PhAAE3     EEAFG.APVLEAYAMTEATHLMCSN..PLPENGP...............HLPGSVGKPVG.QEMAILDENGVVQGPDSKGEVCIRGPNVTK    371
AtAAE3     EEAFG.APVLEAYAMTEATHLMSSN..PLPEEGP...............HKPGSVGKPVG.QEMAILNEKGEIQEPNNKGEVCIRGPNVTK    372
PhAAE14    IEIFPRAKLLSAYGMTEACSSLTFMTLYEPTKESCIQHSHANSSILAHKSDGVCVGKPAPHVEIRIDGD.....DSSSVGRILTRGPHVML    123
AtAAE14    VNIFPCARILSAYGMTEACSSLTFMTLHDPTQES....FKVTYPLLNQPKQGTCVGKPAPHIELMVKLDE....DSSRVGKILTRGPHTML    391

PhAAE13    EYW.....KLPEVTKQSFTDDGYFKTGDTVTVDEDGYYIILGRTNADIMKVGGYKLSALEIEAVLLEHQAIAECCVLGLPDKDYGEVVCAI    526
AtAAE13    EYW.....NLPEVTKESFTEDGYFKTGDAGRVDEDGYYVILGRNSADIMKVGGYKLSALEIESTLLEHPTVAECCVLGLTDNDYGEAVTAI    476
SlAAE13    EYW.....KLPEVTKQSFTDDGYFKTGDTVTVDEEGYYIILGRTNADIMKFGGYKLSALEIEAVLLEHQAILECCVLGLPDKDYGEVVCAI    532
VvAAE13    EYW.....KLPEVTKESFIDGGFFKTGDAVKVDDDGYYIILGRTSADIMKVGGYKLSALEIEAALLEHPAVSECCVLGLPDKDYGEAVCAI    514
OsAAE13    EYW.....RKPEVTAESFIDGGFFKTGDTVTVDDEGYFIILGRTNADIMKVGGYKLSALEIESVLLQHEIVLECAVLGLPDEAYGEIICAI    598
PhAAE3     GYK.....NNPEANKSAFLFG.WFHTGDIGYLDSDGYLHLVGR.IKELINRGGEKISPIEVDAVLVSHPHIAQAVAFGVPDDKYGEEINCA    455
AtAAE3     GYK.....NNPEANKAGFEFG.WFHTGDIGYFDTDGYLHLVGR.IKELINRGGEKISPIEVDAVLLTHPDVSQGVAFGVPDEKYGEEINCA    456
PhAAE14    GYR.....SEMTSKSSSLTSECWLDTGDIGHLDDCGNVWLIGR.SKGRIKSGGENVYPEEVEAVLSQHPGISASVVFGLPDSRLTEMVVAC    208
AtAAE14    RYWGHQVAQENVETSESRSNEAWLDTGDIGAFDEFGNLWLIGR.SNGRIKTGGENVYPEEVEAVLVEHPGIVSAVVIGVIDTRLGEMVVAC    481

PhAAE13    VVPDAEIKRKRGEEL.....KPALTLEELSDWAKER.LASYKIPSRLYLWDS.LPRNAMGKVNKKELKRKLADDPKQV......           597
AtAAE13    IIAESAAKKRREDES.....KPVITLEELCGWAKDK.LAPYKLPTRLLIWES.LPRNAMGKVNKKELKKSLENQE.........           544
SlAAE13    VVPEAEVKRRRDEEL.....KPALTLHELSDWAKEK.LAPYKIPTRLFLWES.LPRNAMGKVNKKELKRKLTEDPK........           601
VvAAE13    IVPEADEKKKQEEEL.....KPAISLEELCTWAKEK.LAPYKLPTQLLLWDL.LPRNAMGKVNKKELKRKLAAEGH........           583
OsAAE13    IVPKEDSKKRAELDS.....KPALTLEALTSWSKDK.LAPYKIPTRLYLWDS.LPRNAMGKVNKKELKKLLGA...........           664
PhAAE3     VIPREGSK...............IDEAEVLRFCKKN.LAAFKIPKRVFMTDS.LPKTATGKIQRRFVAEHFLAQISTAQVPKFG           522
AtAAE3     VIPREGTT...............VTEEDIKAFCKKN.LAAFKVPKRVFITDN.LPKTASGKIQRRIVAQHFLEKP.........           514
PhAAE14    IRLKDSWQWIDSSFHHLVDNGHCLSSTVLQNFCRAKGLTGFKIPKNFVLWKNQFPVTPTGKLRRDQVRAELMSYRQLQPSKL..           290
AtAAE14    VRLQEKWIWSDVENR...KGSFQLSSETLKHHCRTQNLTGFKIPKRFVRWEKQFPLTTTGKVKRDEVRRQVLSHFQIMTSSL..           560
```

**Supplementary Figure S6** Alignment of PhAAE13, PhAAE3 and PhAAE14 with Arabidopsis AtAAE13 (AAM61199), AtAAE3 (NP_190468), AtAAE14 (NP_174340), *Solanum lycopersicum* SlAAE13 (XP_010314289), *Vitis vinifera* VvAAE13 (CBI36114), and *Oryza sativa* OsAAE13 (EEC71525). Dark-gray shading indicates identical residues; light-gray shading indicates conserved substitutions. The alignments were generated using DNAMAN software. Identical and similar amino acids are reversed and shaded, respectively.

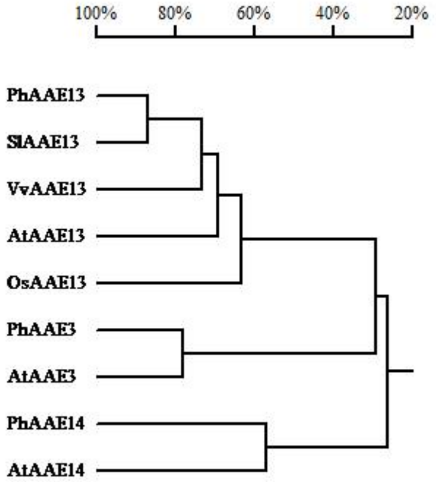


**Supplementary Figure S7** Phylogenetic analysis of Clade VII of AAEs. Petunia PhAAE13, PhAAE3 and PhAAE14 were aligned with *Arabidopsis thaliana* AtAAE13 (AAM61199), AtAAE3 (NP_190468), AtAAE14 (NP_174340), *Solanum lycopersicum* SlAAE13 (XP_010314289), *Vitis vinifera* VvAAE13 (CBI36114), and *Oryza sativa* OsAAE13 (EEC71525) using DNAMAN.
